# Primary Anaplastic-Lymphoma-Kinase-Positive Large-Cell Lymphoma of the Central Nervous System: Comprehensive Review of the Literature

**DOI:** 10.3390/jcm12247516

**Published:** 2023-12-05

**Authors:** Antonio Colamaria, Augusto Leone, Francesco Carbone, Yasser Andres Dallos Laguado, Nicola Pio Fochi, Matteo Sacco, Cinzia Fesce, Francesca Sanguedolce, Guido Giordano, Giorgio Iaconetta, Uwe Spetzger, Luigi Coppola, Elena De Santis, Giulia Coppola, Matteo De Notaris

**Affiliations:** 1 Division of Neurosurgery, Policlinico “Riuniti”, 71122 Foggia, Italy; colamariaa@gmail.com; 2 Department of Neurosurgery, Städtisches Klinikum Karlsruhe, 76133 Karlsruhe, Germany; augustoleone96@gmail.com (A.L.); francesco.carbone615@gmail.com (F.C.); uwe.spetzger@klinikum-karlsruhe.com (U.S.); 3 Faculty of Human Medicine, Charité Universitätsmedizin Berlin, 10117 Berlin, Germany; 4 Faculty of Medicine and Surgery, University of Foggia, 71122 Foggia, Italy; yasserdallos@gmail.com; 5 Division of Neurosurgery, University of Foggia, 71122 Foggia, Italy; 6 Division of Neurosurgery, “Casa Sollievo della Sofferenza”, 71013 San Giovanni Rotondo, Italy; matteosacco88@gmail.com; 7 Hematology Unit, University Hospital, 71122 Foggia, Italy; cinzia.fesce@libero.it; 8 Pathology Unit, Ospedali Riuniti di Foggia, 71122 Foggia, Italy; francesca.sanguedolce@unifg.it; 9 Unit of Medical Oncology and Biomolecular Therapy, Department of Medical and Surgical Sciences, University of Foggia, 71122 Foggia, Italy; guido.giordano@unifg.it; 10 Unit of Anatomy, Pathological Histology and Diagnostic Cytology, Department of Diagnostic and Pharma-Ceutical Services, Sandro Pertini Hospital, 00157 Rome, Italy; giaconetta@unisa.it (G.I.); luigi.coppola@aslroma2.it (L.C.); 11 Department of Anatomical, Histological, Forensic Medicine and Orthopedic sciences, La Sapienza University, 00185 Roma, Italy; elena.desantis@uniroma1.it; 12 Department of Radiological, Oncological and Pathological Sciences, La Sapienza University, 00185 Roma, Italy; coppola1537902@studenti.uniroma1.it; 13 Department of Neurosurgery, University of Salerno, 84084 Salerno, Italy; matteodenotaris@gmail.com

**Keywords:** primary central nervous system lymphoma, anaplastic large-cell lymphoma, ALK, Ki-1, CD-30

## Abstract

Background: Primary anaplastic-lymphoma-kinase (ALK)-positive large-cell lymphoma of the central nervous system (PCNS ALK-positive ALCL) is a rare entity, with a limited consensus reached regarding its management. While this pathology often presents as solitary lesions, the occurrence of multiple tumors within the brain is not uncommon. The lack of distinctive radiological features poses a diagnostic challenge, leading to delays in initiating targeted therapy. Methods: We conducted a comprehensive literature search, identifying seventeen publications for qualitative analysis. Results: The management options and reported patient outcomes in the literature varied significantly, emphasizing the need for a patient-specific approach. The emergence of ALK-specific inhibitors represents a new frontier in this field, demonstrating promising results. Conclusion: PCNS ALK-positive ALCL necessitates a comprehensive understanding and optimized management strategies. A tailored therapeutic approach, integrating surgical intervention with radiotherapy and chemotherapy, appears pivotal in addressing this pathology. The implementation of a therapeutic protocol is anticipated for further advancement in this field.

## 1. Introduction

Primary central nervous system lymphomas (PCNSLs) are a rare entity, constituting less than 1.5% of all intracranial malignancies [1], with an estimated incidence of 1 in 100.000 individuals [2]. Notably, while PNCSLs predominantly affect immunocompromised subjects [3], there has been a recent increase in the incidence among immunocompetent patients [4,5]. B-cell lymphomas are more frequent than their T-cell counterparts, with T-cell lymphomas, including anaplastic large-cell lymphoma (ALCL), accounting for approximately 8.5% of all PCNSLs [1,6]. A subvariant of the T-form is referred to as anaplastic large-cell lymphoma (ALCL), characterized by large pleomorphic immunohistochemical positivity for the CD30 marker [6]. ALCL is an exceptional manifestation in the central nervous system (CNS), typically presenting as a nodal and extranodal disease [1,7] constituting 5% of all human non-Hodgkin lymphomas [4].

The 5th Edition of the World Health Organization’s classification of hematolymphoid tumors delineates two subvariants of ALCL: ALK-positive and ALK-negative forms [8]. Up to 85% of systemic ALCLs exhibit the t(2;5) translocation, where the ALK1 gene on chromosome 2 fuses with the nucleoplasmin (NPM) gene on chromosome 5, resulting in the encoding of the p80 protein, which is strongly implicated in neoplastic degeneration [9]. ALK1 positivity is associated with a younger age at diagnosis and is considered a significant positive prognostic factor [10]. However, despite a generally favorable prognosis, ALK-positive ALCLs can exhibit a rapidly deteriorating clinical course [11].

PCNS ALK-positive ALCLs often manifest as solitary lesions, although multiple brain manifestations are not uncommon. Despite a higher tendency to affect the leptomeninges compared to more common B-cell PCNSLs [12], the absence of pathognomonic radiological features complicates diagnosis, leading to delays in targeted therapy initiation [11].

To date, there is no consensus on treatment protocols for PCNS ALK-positive ALCLs, and management is predominantly empirical, relying on individual institution standards of care [7]. Various therapeutic strategies, including surgical techniques, single or combined chemotherapeutic agents (such as CHOP [7] or the DeAngelis Protocol [13]), and focal or whole-brain radiotherapy, have been reported, with varying efficacies and toxicities [11]. However, diagnostic challenges, the rapid and unpredictable evolution of this malignancy, and the absence of a unified therapeutic protocol [7] contribute to often-unsatisfactory outcomes [4,10,14].

For this article, we conducted a comprehensive review of the literature to compile and critically summarize essential evidence on the clinical manifestations, treatment options, and efficacy rates for PCNS ALK-positive ALCLs.

## 2. Materials and Methods

### 2.1. Search Strategy and Selection Criteria

**Figure 1:**
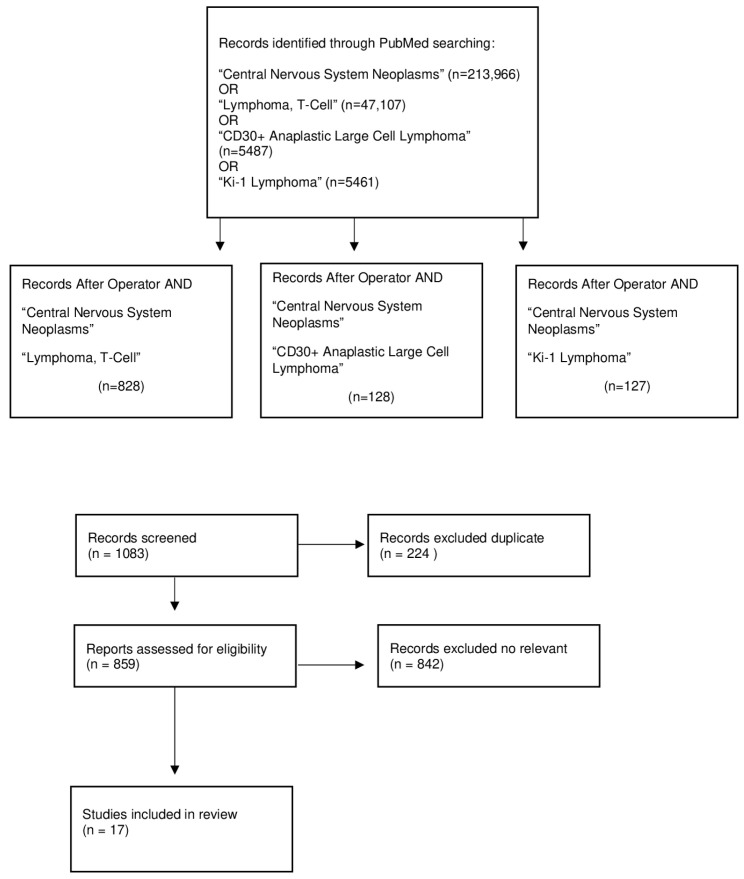
Preferred Reporting Items for Systematic Reviews and Meta-Analyses protocol used for the systematic review, adapted from [15].

A comprehensive search of the literature was performed in compliance with the updated Preferred Reporting Items for Systematic Reviews and Meta-Analyses (PRISMA) 2020 guidelines, as shown in Figure 1 [15]. Article inquiry was operated via the electronic databases MEDLINE/PubMed for manuscripts reporting PCNS ALK-positive ALCLs. Human studies in English published between 1997 and April 2023 were considered for inclusion. Primary search terms included “Central Nervous System Neoplasms”, “Lymphoma, T-Cell”, “CD30+ Anaplastic Large Cell Lymphoma”, and “Ki-1 Lymphoma” in the article titles and abstracts in various MeSH combinations. Inclusion criteria were: (1) PCNS ALCL T-cell lymphomas demonstrating immunohistochemical positivity for the AKL1 marker and (2) availability of sufficient patient-specific clinical, histological, and surgical information. Publications describing meningeal and spinal cord forms were excluded. A total of 1083 records were identified. The extracted citations were then checked for duplicates, and citations of the examined manuscripts were also screened for the purpose of this review. A total of 859 were assessed for eligibility, and 842 were excluded for not complying with the abovementioned criteria through an automated system (Covidence, Veritas Health Innovation, Melbourne, Australia) [16]. One publication was not retrieved with its full text. Finally, seventeen publications were included in the qualitative analysis.

### 2.2. Data Extraction

Two authors (A.Y.D. and F.C.) independently reviewed all abstracts to recognize articles that required full-text review. They investigated abstracts against predefined eligible criteria, and all included studies were discussed with a third author (N.P.F.). The following information was obtained: the author’s name, patient’s age and gender, site of the tumor, clinical presentation, radiological aspects on MRI, liquor characteristics, pathological findings, immunohistochemistry-positive markers, immunohistochemistry-negative markers, ALK gene mutation, treatment, and status at follow-up. Gathered data were stored in a centralized database (Microsoft Excel, Version 16.79.2, Redmond, WA, USA).

### 2.3. Data Analysis

A descriptive analysis of data was performed using mean, median, percentages, and maximum and minimum values. Continuous variables were represented by mean and range values, except in cases otherwise specified. Data were analyzed and processed with SPSS version 24.0.1.1 (14) (IBM, Armonk, NY, USA) and Microsoft Excel (Version 16.79.2, Redmond, WA, USA). Statistical significance was considered for *p*-value < 0.05. Two independent reviewers (A.L. and A.Y.D.) performed the statistical analysis.

## 3. Literature Review

### 3.1. Demographics and Tumor Characteristics

Seventeen cases of ALK-1 positive T-cell brain lymphomas have hitherto been reported (Table 1 and Table 2). The data show a predominantly male prevalence (82.3%, n = 14/17). The median age at diagnosis was 15.9 years, range 2–38. A total of 12 (70.5%) patients presented single lesions, and 5 (29.4%) had multiple foci. Only one tumor affected primarily the cerebellum, one case involved the brainstem (together with the occipital lobe), and one further lesion (5.8%) was located adjacent to the planum sphenoidale; the remaining 14 cases (82.3%) affected exclusively the cerebral hemispheres. Even in the absence of a specific radiological pattern, these lesions tend to show some typically recurring characteristics. For instance, ten cases demonstrated an important contrast uptake—sometimes resulting in a non-homogeneous pattern in T1-weighted images. Among these cases, six lesions demonstrated enhancement in the leptomeninges. In nine cases, a significant perilesional edema was disclosed. Lastly, two lesions showed a peculiar cystic degeneration, and one further caused the erosion of the immediately adjacent bone component. 

### 3.2. Management Algorithms

Treatment options were markedly heterogeneous: for instance, 5.9% (n = 1) were treated with a chemotherapy regimen alone, 23.5% (n = 4) with a combination of chemotherapy and surgical resection, 29.4% (n = 5) with the combination of systemic therapy and radiation therapy, 41.2% (n = 7) with a combination of surgical resection, chemotherapy, and radiation and, in the remaining case, the patient died before the initiation of the treatment (Table 3). Not surprisingly, equally heterogeneous were the outcomes: 55.6% of the cases showed no recurrence of the lesion following the first line of treatment (median follow-up: 54.2 months, range: 13–96); in 29.4% (n = 5) early death (within 6 months of diagnosis) was registered, and in one case (5.9%), the patient was alive at the time of discharge; however, for this last case and two other cases (n = 2; 11.8%) no data were available on survival or the post-treatment course of the disease.

With regard to available chemotherapic options, MTX proved to be the most widely used immunosuppressant agent to be administered, alone or in combination with other drugs or locoregional therapy. It was used as the sole systemic agent in three cases, achieving no evidence of disease ≥ 9 months (max 96 months), while the association with other chemotherapic agents was followed in 10 cases. Other frequently used chemotherapy combinations include the DeAngelis protocol (HD-MTX—with leucovorin rescue, intrathecal MTX, vincristine, procarbazine, and dexamethasone), MATRix (MTX, idarubicin, cytarabine, and thiotepa), BFM90 (prednisone, vincristine, asparaginase, cyclophosphamide, cytarabine, daunorubicin, doxorubicin, methotrexate, and 6-mercaptopurine), CHOP/CHOD (cyclophosphamide, doxorubicin, vincristine, prednisone/dexamethasone), and the VAM Protocol (vincristine, methotrexate—with folinic acid rescue—and cytarabine).

## 4. Discussion

A comprehensive understanding of the physiopathological features inherent to ALK-positive PCNSL remains elusive, primarily owing to the limited number of reported cases and the consequent absence of a standardized management protocol. While diverse treatments have yielded durable complete remissions, the occurrence of intracerebral recurrence is not uncommon [21].

The current diagnostic imaging modalities exhibit insufficiencies in distinguishing PCNSL from other malignant or inflammatory processes, necessitating histology for definitive diagnosis. Despite the rarity of this pathology, it is crucial to consider it among the diagnostic possibilities. The reported data indicate a significant rate of remission, even in the absence of a standardized treatment protocol, underscoring the necessity for early diagnosis and treatment.

The prompt and accurate diagnosis of primary ALCL of the CNS is infrequent. According to a previous study [22], an average of around 40 days is required for diagnosis, hindering early identification and resulting in treatment delays that could prove fatal.

Initially, small lesions discovered along the dura are often misdiagnosed as inflammatory conditions such as meningitis or sarcoidosis. Tuberculosis is commonly considered as the probable infection, because it frequently presents with leptomeningeal enhancement [23].

Before undergoing surgical intervention, a biopsy is advised, to histologically confirm the suspect of PCNSL [24]. However, despite long-standing reports indicating that the extent of surgical removal does not impact the prognosis of this pathology [25], there might be value in attempting maximal tumor resection when symptoms of increased intracranial pressure manifest due to the mass effect of the tumor [23].

Despite sharing histologic, immunophenotypic, and clinical features with extra-CNS ALCL, PCNS ALK-positive ALCL demonstrates a more aggressive clinical behavior [14]. Favorable prognostic factors in ALCL include youth, unifocal tumor presentation, and the absence of necrosis. Conversely, older age, multifocal tumor presentation, and extensive necrosis correlate with an elevated risk of mortality [9]. Notably, the expression of CD56, a neural cell-adhesion molecule, in ALCL is associated with a poorer overall prognosis, increased recurrence, CNS involvement, and a higher likelihood of bone involvement [14,26]. Despite its rarity, CD56 positivity has been observed in only two cases in the literature.

PCNS ALK-positive ALCL is a rare entity, accounting for less than 4% of all PCNSL cases in Western countries [27,28]. Due to the scarcity of this tumor, no consensus exists on its management, and treatment approaches are typically empirical, although methotrexate (MTX) monotherapy is often associated with improved survival rates [2]. While chemotherapy has demonstrated optimal outcomes, most patients necessitate combined protocols, involving locoregional therapy [2,29]. For instance, favorable outcomes have been achieved through the combination of corticosteroid therapy and radiation [30]. Considering the uncertainty surrounding the efficacy of additional combined-modality protocols in improving survival and reducing delayed neurotoxic effects, radiation may be a rational choice in younger patients, those with residual or recurrent tumors, and those with inadequate responses to chemotherapy [21,31].

Given the infrequency of PCNS ALK-positive ALCL, the reported treatment protocols exhibit substantial prognostic variability. However, with the evolution of personalized medicine, particularly the development of ALK-specific inhibitors, the molecular mechanisms governing tumorigenesis have become targets for more effective therapeutic approaches [32]. Recent research underscores the efficacy of ALK inhibitors in relapsed or refractory ALK-positive ALCL, demonstrating reduced toxicity. While first-generation ALK inhibitors like crizotinib have limitations in CNS penetration, next-generation inhibitors such as alectinib, brigatinib, ceritinib, and lorlatinib show promise in crossing the blood–brain barrier [33]. Several studies highlight the clinical significance of these next-generation ALK inhibitors in relapsed or refractory cases [34,35,36]. However, further research is warranted to establish their efficacy in primary settings, considering the common adverse events of gastrointestinal toxicities, elevated liver enzymes, and fatigue [33]. 

## 5. Conclusions

The lack of consensus regarding the management of PCNS ALK-positive ALCL impedes standardized therapy for this rare tumor, potentially leading to unfavorable outcomes. The emergence of targeted monoclonal therapies is anticipated to mitigate inter-institutional differences in adopted management algorithms. Nevertheless, given the extreme paucity of similar cases, this report of experiences may facilitate a more standardized and evidence-based therapeutic approach. Further comprehensive studies are envisaged to optimize our therapeutic armamentarium in cases of PCNS ALK-positive ALCL.

## Figures and Tables

**Table 1:** Demographic, topographical, clinical, and radiological characteristics of PCNS ALK-1 positive ALCLs. George et al. [9] reviewed data from 12 previously published cases and investigated tissue samples of five of these which have been made available to the author for further immunopathologic study. Clinical histories and presentations, radiologic imaging, details of treatment, and outcomes were obtained via contact with clinicians and/or the submitting pathologists. M: male. F: female. GE: gadolinium enhancement. CT: computed tomography.

Case Number	Reference	Age/Gender	Site	Symptoms	Radiological Aspect of MRI
1	Ponzoni, M. et al. [17]	29/M	Single Cortical–subcortical fronto-temporal lobe left	Fever, cephalgia, epileptic activity	Lesional, pial, and subarachnoid GE. T2 hypo-isointense. Perilesional edema (on admission)
2	Nomura, M. et al. [6]	20/M	Single Left frontal lobe	Epileptic activity (generalized seizures)	Lesional GE. High-intensity signal on T2. Edema and midline shift
3	Geetha, N. et al. [10]	19/M	Single Right cerebellar hemisphere	Cephalgia, emesis, obstructive hydrocephalus	Well-defined lesion. T1 hypointense. T2 iso-hyperintense. Perilesional edema
4	Kuntegowdenahalli, L. et al. [3]	18/M	Single Left parieto-occipital lobe	Fever, cephalgia, epileptic activity (seizures)	Hyperintense on T2-Flair. Midline shift
5	Splavski, B. et al. [2]	26/M	Single Partial intraventricular, frontal horn of the left lateral ventricle	Incidentaloma	GE. Partial cystic degeneration. Perifocal edema. Subependimal intra-axial spread. Iso-iperintense on T2-images
6	Liu, Q. et al. [14]	12/M	Single Right occipital lobe and falx cerebrum	Cephalgia, emesis	GE. Partial cystic degeneration. Perilesional edema. Midline shift
7	Abdulkader, I. et al. [4] Reviewed by George, D. H. et al. [9]	13/M	Multiple Right parietal lobe and right frontal lobe	Cephalgia, emesis	T1 hypointense, T2 hyperintense signal. Perilesional and leptomeningeal GE
8	George, D. H. et al. [9]	18/F	Single Left temporal lobe and surrounding dura	NA	NA
9	Karikari, I. O. et al. [12]	4/M	Multiple Bilateral frontal lobe and pineal region	Epileptic activity (tonic–clonic seizures), Fever, cephalgia, emesis, nuchal rigidity	Leptomeningeal and lesional GE. T1 hypointense and T2 hyperintense
10	Ozkaynak, M. F. et al. [18]	9/M	Multiple Bilateral frontal lesions extended into the superior frontal gyri	Fever, epileptic activity (focal seizures)	Meningeal GE. MR spectroscopy: elevated choline, decrease N-acetilaspartate, inverted lactate peak
11	Shah, A. C. et al. [5]	2/M	Single Right cerebral hemisphere and surrounding leptomeninges	Lethargy, hemiparesis, epileptic activity	Edema. Uncal herniation. Midline shift
12	Furuya, K. et al. [11]	11/M	Multiple Left parietal lobe	Cephalgia, nausea	Focal meningeal GE, edema. Midline shift
13	Rupani A. et al. [19]	17/M	Single Right fronto-parietal lobe	Cephalgia, epileptic activity, left arm paresis	Well-circumscribed lesion presenting GE. Skull bone erosion. Scalp swelling
14	Vivekanandan, S. et al. [7]	20/M	Single Right silvian fissure	Epileptic activity	Peripheric GE
15	Havlioglu, N. et al. [1] Reviewed by George, D. H. et al. [9]	4.5/F	Multiple Left occipital lobe and left brain stem	Cephalgia, nausea, emesis, nuchal rigidity, fever	Multiple densities scattered over the brain surface and brain stem. CT: lesions in the cervical and lumbar segments of the spinal cord
16	Buxton, N. et al. [20] Reviewed by George, D.H. et al. [9]	10/F	Single Right parietal lobe abutting against the falx	Leftsided sensory disturbance, hemiparesis, cephalgia	Irregular, heterogeneous mass. Minor falcine GE
17	Carmichael, M. G. et al. [13]	38/M	Single Intraparenchymal parieto-occipital right	Epileptic activity, syncope, left-sided hemiparesis, visual field deficit, ataxia	Surrounding edema. Midline shift

**Table 2:** The main CSF and histo-pathological features of the lesions examined are summarized here. With regard to the CSF, the parameters that were most frequently altered were the leucocyte count, glicorrachia, and protidorrachia. Histological analysis showed recurrent alterations in most of the cases. Lastly, the immunohistochemical study showed great heterogeneity in the expression of markers. GCSF: granulocyte colony-stimulating factor. LCA: leucocyte common antigen. EMA: epithelial membrane antigen. GFAP: glial fibrillary acidic protein.

Case Number	Microscopic Analysis of CSF	Pathological Findings	Immunohistochemistry-Positive Markers	Immunohistochemistry-Negative Markers	ALK Gene Mutation
1	Clear, colorless 5 lymp/mm^3^ Protein 53 mg/dL No malignant cells Negative cultures	Medium-to-large lymphoid-looking cells. Kidney-shaped nuclei, prominent nucleoli, abundant cytoplasm. Few “hallmark cells”. Apoptotic figures, no necrosis. Infiltrate of macrophages, granulocytes, and small lymphocytes	ALK-1, LCA, CD30, EMA, monoclonal CD3 and CD45RO	CD20, CD79a, S-100 protein, GFAP, myeloperoxidase, CD34, CD68 (KP-1)	NA
2	NA	Large, atypical lymphocytes containing scattered horseshoe-shaped nuclei	ALK-1, CD3	CD20	NA
3	NA	Sheets of pleomorphic tumor cells with classical doughnut cells	ALK, LCA, CD30	CD5, CD20	NA
4	No malignant cells	Suggestive of ALCL	ALK, LCA, CD30, CD4	CD3, CD7	NA
5	NA	Polymorphous cells with hyperchromatic nuclei. Sporadic mitosis	ALK, Vimentin, CD45LCA, EMA, CD3, CD4, CD30, CD99, MUM-1, Ki67 75%	Cytokeratin AE1/AE3, cytokeratin MNF116, TTF-1, PLAP, HMB45, GFAP, keratin 7, keratin 20, CD20, CD10, CD8, bcl2, bcl6, NSE, Tdt	NA
6	NA	Lymphoid cells with a diffuse monotonous growth pattern with focal or sheet necrosis and starry-sky mimicking. Irregular-shaped nuclei with multiple basophilic nucleoli. Abundant pale or basophilic cytoplasm. Prominent mitosis	ALK1, CD30, Granzyme B, TIA-1, CD56, MUM-1, EMA, CD4 Ki67 95%	CD2, CD3, CD5, CD7, GFAP, PLAP, CD34, CD45, CD20, CD79a, TdT, CD99, BCL-2, BCL-6, CD10	Monoclonal TCRγ gene rearrangement Gene translocation involving ALK
7	1450 WBC/μL Glucose 34 mg/dL Protein 135 mg/dL Atypical lymphocytes with eccentric-shaped nuclei, prominent nucleoli, scant dense cytoplasm, multiple cytoplasmatic vacuoles. Binucleation. Mitotic figures	Large cells with amphophilic cytoplasm, large nuclei (often horseshoe-shaped) with prominent nucleoli. Focal necrosis, lymphoplasmacytic infiltrate. High mitotic rate. Atypical mitotic figures	ALK1, CD30, LCA, UCHL1, P80, EMA CD3, CD45RO (by George, D. H. et al. [9])	Cytokeratins, KP-1, B-cell markers	NA
8	NA	Necrosis absent	ALK1, CD45RO	B-cell markers	NA
9	Elevated WBC Glucose: low Protein levels: increased	Large, atypical cells with irregular nuclei with a moderate amount of eosinophilic and basophilic cytoplasm. No Reed–Sternberg-type cells	ALK-1, CD30, CD7	PLAP, human chorionic gonadotropin, a-fetoprotein, keratin, NFP, NEU-N, synaptophysin, S-100 protein, CD1A	Balanced reciprocal translocation between crom. 2 and crom. l5 with breakpoints at bands 2p23 and 5q35
10	In total, 27 WBC (63% lymphocytes, 31% monocytes, 6% neutrophils) Negative Gram stain and culture Flow cytometry: abnormal CD8-positive T-cell population	Large angiocentric cells invading the parenchyma. High mitotic rate	ALK-1, LCA, CD3, CD8, CD30, Ki-67 Flow cytometry CSF: CD2, CD7	CD5, CD20, CD79a, TDT, SYN, NF, GFAP Flow cytometry CSF: loss of pan T-cell markers CD3 and CD5, CD56, CD57, TdT	NA
11	NA	Multinodular, pleomorphic large cells with dural infiltration. Large mononuclear and binuclear atypical cells. Vascular/endothelial proliferation with congestion, focal hemorrhage, and broad necrosis. Scattered mitoses	ALK-1, CD30 (Ki-1), CD43	EMA, S-100 protein, CD1a, CD3, CD20, CD15 (Leu-M1), GFAP, placental alkaline-phosphatase, muscle-specific actin, desmin	NA
12	Glucose 70 mg/dL, protein 130 mg/dL, cell count 237 cells/mm^3^ with a differential count of 68% polymorphonuclear cells No malignant tumor cells (on CSF cytology)	Large, polymorphic tumor cells, diffusely infiltrate throughout the cortex. Pleomorphic nuclei, prominent nucleoli, abundant clear or eosinophilic cytoplasm. No bacteria	ALK-1, EMA, LCA, CD30 (Ki-1)	GFAP, CD3, UCHL-1 (CD45RO), CD20, CD79, KP-1 (CD68)	NA
13	NA	Large pleomorphic cells with abundant eosinophilic-to-amphophilic cytoplasm and prominent nucleoli. Necrosis absent	ALK1, CD30, CD43, LCA, EMA	Myeloperoxidase, chloroacetate esterase	NA
14	Unremarkable	Sheets of large cytologically atypical lymphoid blast cells interspersed with frequent neutrophil polymorphs. Vesicular nuclei and prominent nucleoli, relatively abundant amphophilic cytoplasm	ALK, CD3, CD30	NA	NA
15	Total of 90 RBC/μL, 10 WBC/μL with large, atypical lymphocytes Glucose 51 mg/dL Protein 210 mg/dL	Large cells with amphophilic cytoplasm, large nuclei with prominent nuclear membrane irregularities, and prominent nucleoli. Focal necrosis and lymphoplasmacytic infiltrate. High mitotic rate, atypical mitotic figures	CD30, EMA ALK-1 (by George, D. H. et al. [9]) CSF cytology: large, atypical lymphocytes with eccentric oval-shaped nuclei, prominent nucleoli, scant dense cytoplasm containing multiple cytoplasm vacoles. Binucleation, mitotic figures	LCA, cytokeratin, neuron-specific enolase, KP-1, B-markers, T-markers, monocyte/macrophage markers. Cytometric analysis CSF: no aberrant pan-T surface marker expression	No monoclonal rearrangement of T beta receptor, K or lambda light-chain genes, or immunoglobulin heavy-chain locus
16	NA	High mitotic rate. High level of apoptosis and an unusual pattern of spread Necrosis (by George, D. H. et al. [9] )	Ki-1 AKL1, CD43, CD45RO (by George, D. H. et al. [9])	B-markers (by George, D. H. et al. [9])	NA
17	NA	Malignant cells consistent with ALCL	AKL-1, CD30, CD45,	NA	NA

**Table 3:** Here, the treatment protocols and patients’ outcomes are summarized. Surgical therapy, in combination with chemotherapy and/or radiotherapy regimens, was employed in ten cases: a gross-total resection (GTR) was achieved in five of these. Radiotherapy was administered in eleven patients and, in five cases, was associated with a combination of one or more chemotherapy regimens, while, in the remaining six, radiotherapy was used in conjunction with chemotherapy and surgical intervention. Equally variable are the individual application regimens: in seven out of twelve cases, radiotherapy was applied to the whole brain (in two cases, with extension to the spinal cord, as well), and in two, it had focal administration, consistent with the site of the lesion, while in the remaining two cases, the type of administration was not specified. CHT: chemotherapy. RT: radiotherapy. GTR: gross total resection. STR: subtotal resection. HD-MTX: high-dose methotrexate. NED: no evidence of disease.

Case Number	Treatment	Status at Follow Up
1	Biopsy CHT: MATILde regimen (MTX, idarubicin, cytarabine, thiotepa) RT: Whole-brain RT	NED at 13 months (from completion of the treatment)
2	GTR CHT: HD-MTX	NED at 5 years
3	STR CHT: BFM90 ALCL Protocol	Recurrence 9 months after surgery Exitus a month later
4	GTR CHT: DeAngelis protocol (HD-MTX, leucovorin, Intrathecal—MTX via lumbar puncture, vincristine, procarbazine, Dexamethasone). Cytarabine (after RT) RT: Whole brain	On prophylactic antiepileptic medication (no further info regarding OS or PFS available)
5	GTR CHT: HD-MTX, HD-Cytarabine. GCSF, folinic acid (leucovorin) RT: Whole brain	NED at 2 years
6	Biopsy	Exitus in one month
7	Biopsy CHT: vincristine, Etoposide, MTX, cyclophosphamide, dexamethasone, cytarabine	Exitus shortly after CHT treatment
8	CHT RT (local or whole brain not specified)	NED at 5.2 years
9	Biopsy CHT: doxorubicin, prednisone, vincristine. RT: Craniospinal	Alive at discharged (for completion of chemotherapy and radiation therapy)
10	STR CHT: Dexamethasone, HD-MTX, etoposide, BCNU. Intraventricular MTX, hydrocortisone, Ara-C RT: Focal	NED at 26 months
11	STR CHT: HD-MTX	NED at 8 years (from therapy completion)
12	Methylprednisolone for ICP before diagnosis STR via biopsy CHT: HD-MTX RT: Whole brain	NED at 8 years after completion of treatment
13	Biopsy CHT: Steroids, cyclophosphamide, adriamycin, vincristine RT (not specified)	Exitus after 1 month
14	GTR CHT: CHOD (cyclophosphamide, doxorubicin, vincristine, dexamethasone, allopurinol), BCNU (carmustine), VAM (vincristine, MTX, folinic acid, cytarabine) RT: focal	NED at 8 years from initial presentation
15	STR via Biopsy CHT: CHOP Protocol (cyclophosphamide, doxorubicin, oncovin, prednisone)	Gradually improved with supportive therapy (no further info regarding OS or PFS available)
16	Dexamethasone (before diagnosis) GTR CHT: United Kingdom Children’s Cancer Study Group 9003 protocol (HD-MTX, cyclophosphamide, daunorubicin, cytosine, vincristine, prednisolone) RT: craniospinal	Exitus after 6 months
17	Biopsy HD-dexamethasone, phenytoin (before diagnosis) CHT: HD-MTX/leucovorin, vincristine, procarbazine, dexamethasone. Intrathecal MTX (Ommaya reservoir). HD-systematic cytarabine (according to the DeAngelis protocol) RT: Whole brain (before and after diagnosis)	NED at 15 months following therapy

## Data Availability

No new data were created or analyzed in this study. Data sharing is not applicable to this article.
